# The Usefulness of Mandibular Anglectomy in Patients With Masticatory Muscle Tendon-Aponeurosis Hyperplasia: A Preliminary Single-Center Survey

**DOI:** 10.7759/cureus.74994

**Published:** 2024-12-02

**Authors:** Daigo Yoshiga, Sho Mitsugi, Taishi Otani, Hiroki Tsurushima, Osamu Sakaguchi, Manabu Habu, Izumi Yoshioka

**Affiliations:** 1 Department of Science of Physical Functions, Division of Oral Medicine, Kyushu Dental University, Kitakyushu, JPN; 2 Second Department of Oral and Maxillofacial Surgery, Osaka Dental University, Osaka, JPN; 3 Department of Science of Physical Functions, Division of Oral and Maxillofacial Surgery, Kyushu Dental University, Kitakyushu, JPN

**Keywords:** anglectomy, masticatory muscle tendon-aponeurosis hyperplasia, mmtah, square mandible, trisumus

## Abstract

Objective

Masticatory muscle tendon-aponeurosis hyperplasia (MMTAH) is a recently identified condition characterized by restricted mouth opening due to hyperplasia of the temporalis muscle tendon and masseter muscle aponeurosis. This study examines the treatment and clinical course of patients with MMTAH who underwent surgery at our hospital.

Subjects and methods

The study included 14 patients (four males and 10 females; mean age: 33.9 years; age range: 17-50 years) who were clinically diagnosed with MMTAH at our hospital between 2008 and 2020. The surgical procedure, as well as the preoperative, intraoperative, and postoperative mouth opening range of each patient, were examined.

Results

In all cases, the mouth opening range significantly improved postoperatively. Patients who underwent mandibular anglectomy showed a significantly greater mouth opening range both preoperatively and postoperatively compared to those who did not undergo the procedure. Additionally, mandibular anglectomy was effective in maintaining the mouth opening range at six months in patients who underwent the procedure, in contrast to those who did not.

Conclusions

Mandibular anglectomy appears to be effective in maintaining the mouth opening range in patients with MMTAH. While additional case studies are needed to confirm our findings, we believe that mandibular anglectomy is a valuable treatment option for MMTAH.

## Introduction

Masticatory muscle tendon-aponeurosis hyperplasia (MMTAH) is a relatively new condition characterized by limited mouth opening due to hyperplasia of the temporalis muscle tendon and masseter muscle aponeurosis. The etiology of MMTAH remains unknown, and the clinical manifestations are not fully understood. Furthermore, no established treatment guidelines exist for this condition. Until recently, MMTAH has often been misdiagnosed as a temporomandibular disorder [1]. Many oral surgeons and dentists remain unaware of MMTAH, increasing the risk of it being confused with other disorders, such as temporomandibular joint (TMJ) disorders. Some patients primarily report reduced TMJ mobility, although the restricted range of motion can arise from various causes, including TMJ ankylosis, articular disc abnormalities, systemic diseases, and tumors [2]. Difficulty opening the mouth is a common symptom of joint hypermobility, which is diagnostically challenging as clinicians must rule out other potential causes before reaching a final diagnosis. Recently, MMTAH has been added to the list of TMJ hypermobility disorders.

MMTAH involves a limitation in mouth opening due to impaired muscle advancement resulting from hyperplasia or structural changes in the tendons of masticatory muscles, primarily the masseter, temporalis, and internal pterygoid muscles. Initially reported by a Japanese group, MMTAH has since gained increasing recognition worldwide [3]. Although the exact cause of MMTAH is still unclear, some studies suggest that extrafunctional habits such as bruxism and teeth grinding may be risk factors, contributing to tendon and tendon membrane hyperplasia and muscle lengthening disorders [4-6]. Typical characteristics of MMTAH patients include a square-shaped mandible with hyperplasia of the mandibular angle, masseter muscle tendon membrane hyperplasia, temporalis muscle tendon membrane hyperplasia, and limited mouth opening [1,3].

Restricted mouth opening is a significant health concern as it impedes eating, complicates oral cavity examination, and can increase mortality risk during emergency intubation [3]. Caution is also required during general anesthesia, as patients with masticatory muscle tendon hyperplasia may experience further difficulty opening the mouth after muscle relaxants are administered. Nasotracheal intubation is recommended to secure the airway. This approach has become standard in surgeries for MMTAH at our hospital to maintain optimal surgical field visibility and check occlusal relationships. Anesthesiologists should be prepared for potential difficulties in intubating such patients [7].

MRI is a valuable diagnostic tool for MMTAH, allowing visualization of the tendons and periosteum. On horizontal MRI scans, an overhang of the masseter muscle is often visible along the anterior margin of the mandibular ridge. Palpation of the oral cavity near the anterior margin of the masseter muscle at maximum mouth opening may reveal a hard, cord-like structure, which corresponds to the MRI’s stripe root appearance, aiding in diagnosis [8]. Hypertrophy of the masseter muscle and a square-shaped mandible are common features in patients with mouth opening disturbances. Murakami et al. reported that early intervention, including masseter muscle tendonectomy, resection or dissection of the coronoid process, and occasionally mandibular angle resection, can improve symptoms [9]. Their study suggested that both the masseter and temporalis muscles contribute to dysostosis, and they also noted potential involvement of the medial pterygoid muscle. Surgical treatments have been reported as effective, although consensus on management strategies remains lacking [10].

Recent studies have identified genetic factors that may contribute to MMTAH. Protein levels of ankyrin repeat domain 2, troponin T1, and myosin heavy chain 7, associated with slow muscle fibers and mechanical loading, are highly expressed in patients with MMTAH. Gene expression patterns in these patients appear age-related and similar, suggesting that mechanical loading may play a role in MMTAH development [11]. Additionally, whole genome sequencing of 10 MMTAH patients and their families has implicated PCDH1 and BAIAP3 as potential causative genes for the condition [12]. Therefore, the genetic underpinnings of MMTAH are gradually becoming clearer.

Histopathological analysis of severed tendons and muscles has revealed calcified nodules containing silicon, calcium, and phosphorus, which may account for the sounds heard when tendons are surgically severed [13]. Fatty infiltration was observed in the masseter muscle bundles of younger patients, suggesting disuse atrophy, while mild degenerative changes, including tendon calcification, were noted in patients aged 40-50. The thickening of the masticatory muscle tendons and tendon membranes in MMTAH is thought to result from hyperplasia [9]. Although much about MMTAH remains unclear, this report examines patients who underwent surgical treatment at our hospital and further investigates the outcomes of surgery with and without mandibular anglectomy.

## Materials and methods

Subjects

We retrospectively studied 14 patients who visited our hospital and were clinically diagnosed with MMTAH, with no abnormalities in the TMJ, between October 2008 and May 2020. The surgical procedure was explained to all patients. The mouth opening range (measured between the upper and lower central incisors) was assessed preoperatively, intraoperatively, and postoperatively for each case (Table 1).

**Table 1:** Summary of the MMTAH cases MMTAH, masticatory muscle tendon-aponeurosis hyperplasia

Case	Age (year)	Sex	Incisal edge distance (mm)	Aponeurectomy of the masseter muscle	Resection of the mandibular process	Resection of the mandibular angle	Incisal edge distance (mm)		
			Pre-operation				Intraoperative	One month post-operation	Six months post-operation
1	20	M	22	Performed	Performed	Unperformed	30	24	22
2	26	F	24	Performed	Performed	Unperformed	50	28	20
3	24	F	24	Performed	Performed	Unperformed	40	35	35
4	24	F	23	Performed	Performed	Unperformed	45	30	35
5	34	M	24	Performed	Performed	Unperformed	46	36	38
6	41	M	22	Performed	Performed	Unperformed	45	38	48
7	50	F	26	Performed	Performed	Unperformed	53	40	43
8	44	F	22	Performed	Performed	Unperformed	60	31	43
9	39	F	27	Performed	Performed	Unperformed	55	38	44
10	17	M	25	Performed	Performed	Performed	61	44	50
11	35	F	27	Performed	Performed	Performed	48	40	41
12	38	F	15	Performed	Performed	Performed	60	36	53
13	48	F	25	Performed	Performed	Performed	52	40	48
14	35	F	27	Performed	Performed	Performed	55	36	42

Treatment

Preoperatively, we assess whether mouth opening training could improve the mouth opening range. While some cases show improvement with mouth opening exercises alone, this is not a fundamental treatment, and regression may occur, or satisfactory mouth opening may not be achieved. To maintain the mouth opening range, postoperative opening training is crucial. Since the prognosis depends on effective opening training, we emphasize the importance of mouth opening exercises before surgery to make patients aware of the need for postoperative training and to teach them how to perform the exercises themselves.

In the 14 cases treated at our hospital, temporalis tendonotomy was performed in all 14 cases, masseter muscle tendonectomy in all 14 cases, and mandibular anglectomy in five cases. These surgeries were all conducted via an intraoral approach. The primary goal of resection and dissection of the coronoid process, as well as dissection of the temporalis tendon for this condition, is to remove the temporalis tendon from the coronoid process. Therefore, we refer to these combined procedures as temporalis tendonotomy in this report.

**Figure 1:**
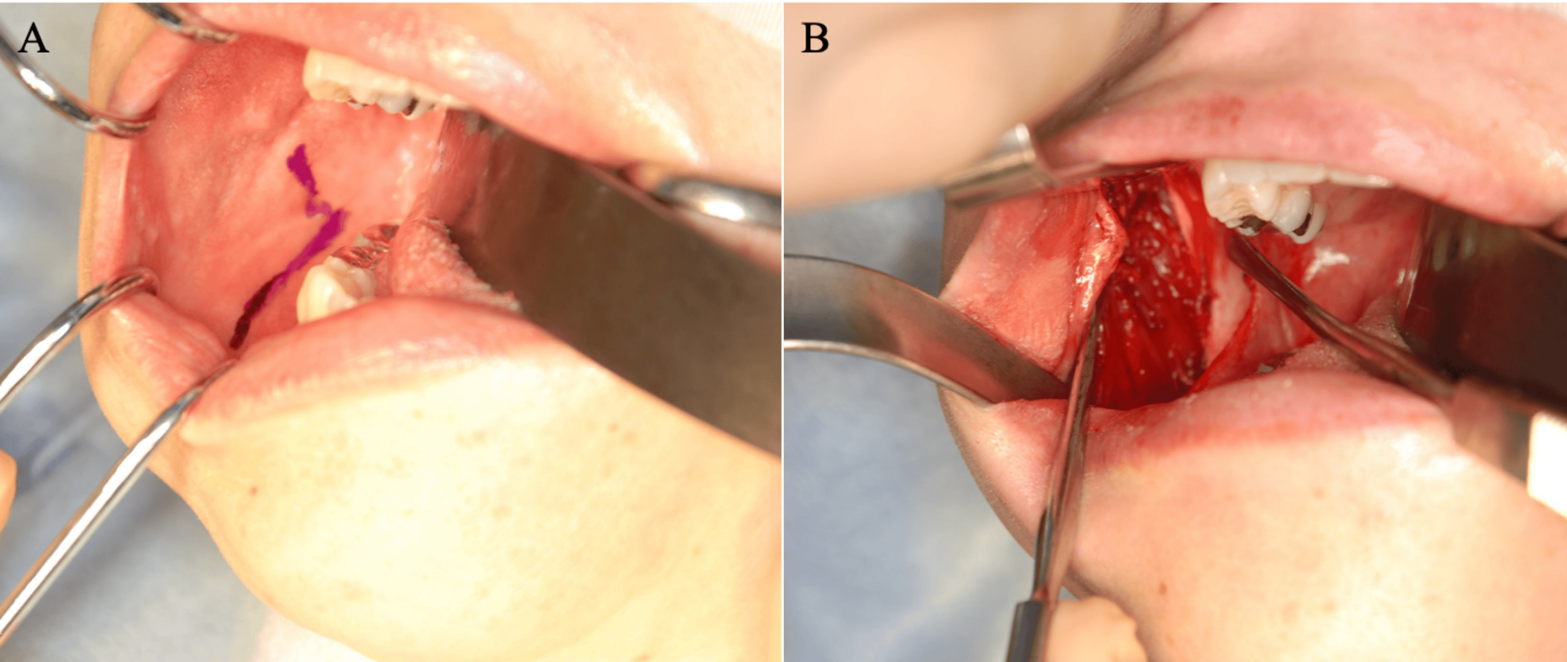
Intraoperative findings (intraoral approach) (A) A flying bird-like mucosal incision was made along the external oblique line of the mandibular branch, extending from the upper anterior margin of the mandibular branch to the first mandibular molar. (B) White aponeurosis on the lateral surface of the left masseter muscle, visible after dissection.

The surgical procedure performed at our hospital is as follows: first, general anesthesia with nasal intubation is administered, as trismus is common in MMTAH patients. The procedure begins with a masseter muscle tendonotomy. A flying bird-like mucosal incision is then made along the external oblique line of the mandibular branch, from the upper anterior margin of the mandibular branch to the first mandibular molar. The mucoperiosteum is dissected to expose the masseter and temporalis muscles attached to the mandibular branch. Next, the anterior margin of the outermost aspect of the masseter muscle is exposed beneath the masseter fascia, extending from near the inferior border of the zygomatic arch to the inferior border of the mandible. The masseter tendon membrane is then exposed from the anterior margin of the masseter muscle to the lateral aspect of the masseter muscle. While the anterior margin of the masseter muscle is muscular, dissection posteriorly reveals a shiny white tendon membrane on the outermost surface of the masseter muscle (Figure 1).

**Figure 2:**
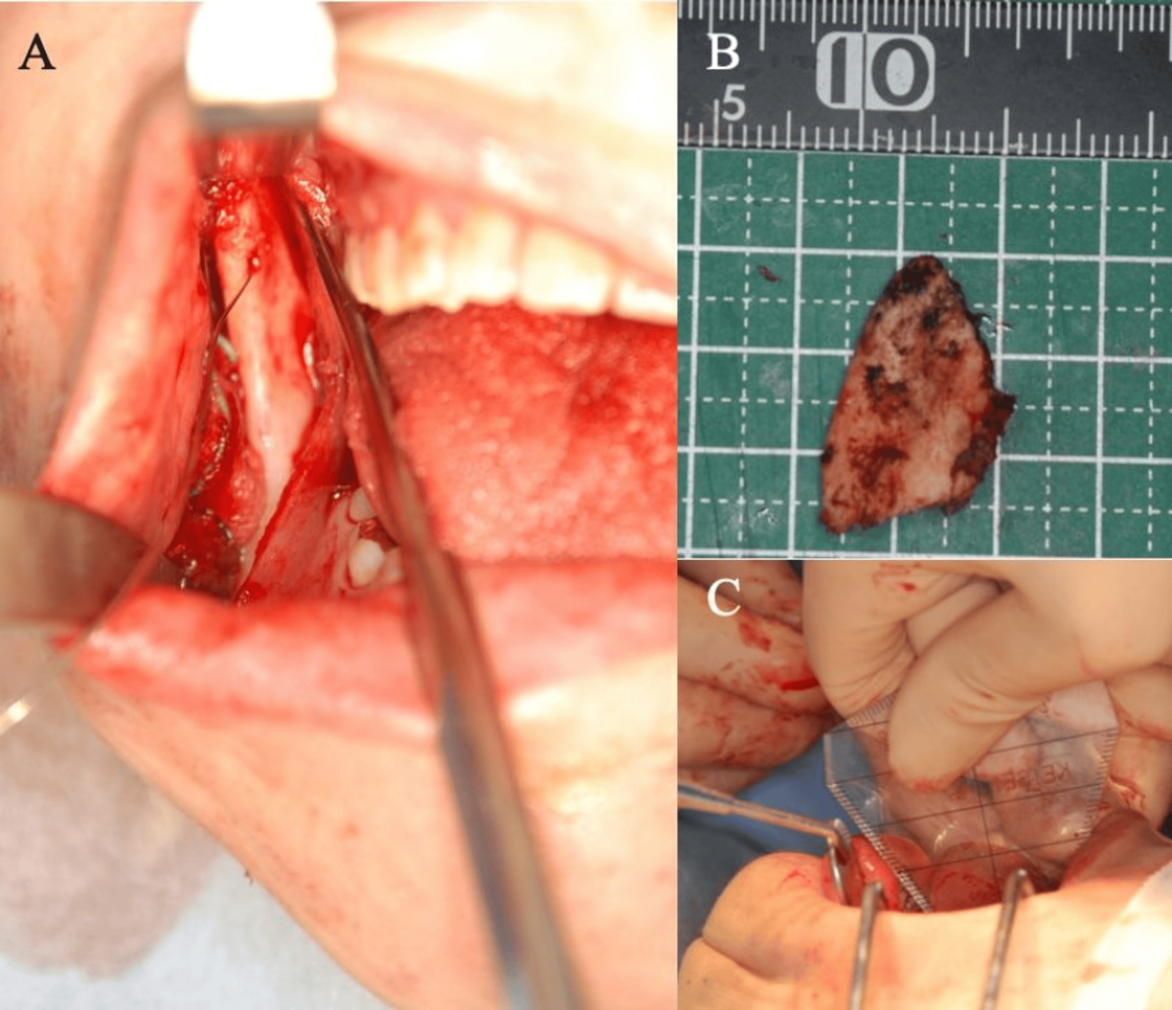
Coronoidectomy (A, B) The muscle process was cut and extracted using a reciprocating bone saw or ultrasonic bone scalpel. (C) The surgery continued while the mouth opening range was monitored intraoperatively, with the range improving from 30 mm to 40 mm.

The tendon membrane is separated from the muscle, extending from near the inferior border of the zygomatic arch to the inferior border of the mandible, and is then removed to assess the mouth opening range. Following this, a temporalis tendonotomy is performed. The mucosa of the anterior mandibular branch is elevated along with the cheek muscle, exposing the temporalis muscle attached to the anterior border of the mandibular branch. Typically, the temporalis muscle that attaches to the coronoid process from the anterior border of the mandibular branch consists of both muscle and tendon. The portion of the temporalis muscle that stops at the anterior border of the mandibular branch is primarily a solid white tendon with minimal muscle. In some cases, the temporalis muscle extends from its attachment at the muscular process to the anterior border of the mandibular branch near the posterior molar triangle. These adhered tendons are dissected or detached, and the muscle process is further cut and extracted using a reciprocating bone saw or an ultrasonic bone scalpel (Figure 2).

**Figure 3:**
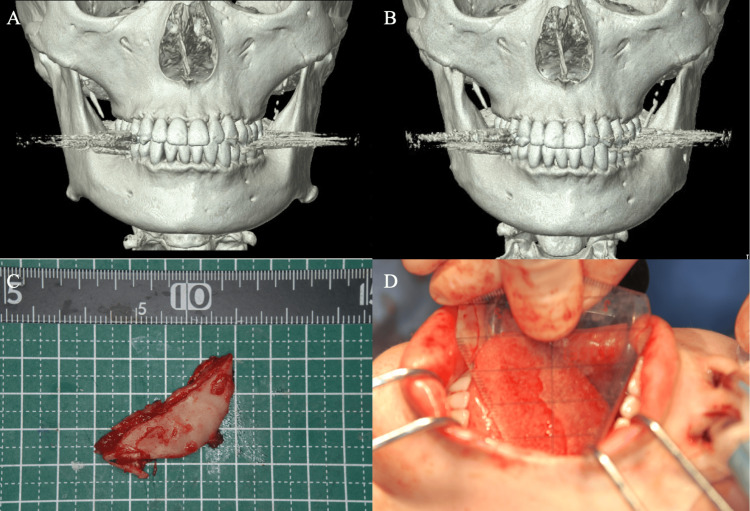
Mandibular anglectomy (A) Preoperative 3D CT image. (B) Postoperative 3D CT image. (C, D) Resection of the mandibular angle. The mandibular angle was resected equally on both sides using an oscillating saw blade. Intraoperative mouth opening change: from 40 mm to 60 mm.

In some cases, temporalis tendonotomy may be limited to dissection without the resection of the elongated coronoid process. If there is no overgrowth of the coronoid process, it is important to determine whether dissection of the temporalis tendon or coronoid process should be performed instead of resecting the coronoid process. Intraoperatively, the mouth opening range should be reassessed after this procedure. Based on a growing body of cases, we have recently concluded that if a sufficient mouth opening of 45 mm is achieved, the surgery can be considered complete. However, if the mouth opening range remains insufficient, mandibular anglectomy is performed as the final step of the procedure (Figure 3).

This procedure is primarily indicated to address masseter muscle hypertrophy and associated mandibular angle hyperplasia. Its goal is to detach the masseter and medial pterygoid muscles from the mandibular branch and reduce the excessive mandibular angle. This allows the muscles to be reattached in a more functional state, improving muscle extensibility. Additionally, the reduction of the mandibular angle decreases the resistance of the surrounding tissues when opening the mouth, thereby increasing the mouth opening range.

**Figure 4:**
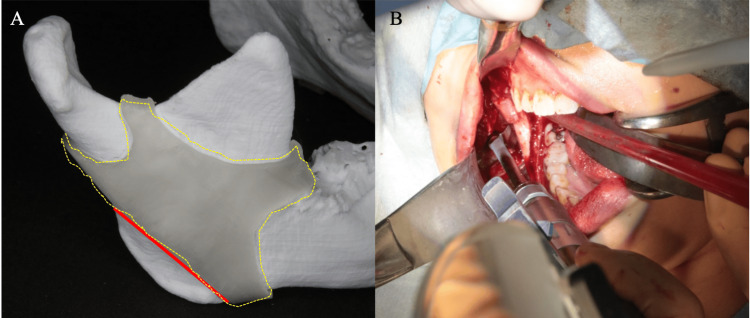
Patient-specific 3D-printed cutting guides (A) Cutting guide derived from a 3D model (yellow dotted line), with the osteotomy line indicated by the red line. (B) Intraoperative findings, showing the application of the cutting guide during the procedure.

This step is considered a last resort, as it requires a certain degree of mouth opening, achievable through the intraoral approach. During the procedure, the masseter muscle attached to the external surface of the mandibular branch, as well as the masseter and medial pterygoid muscles attached to the inferior and posterior borders of the mandible, are dissected. The excessively protruding mandibular angle is then removed using reciprocating and oscillating bone saws. Recently, our hospital has adopted a cutting guide based on a 3D model derived from preoperative CT data. This approach allows for safer and more accurate osteotomy, guided by preoperative simulation (Figure 4).

**Figure 5:**
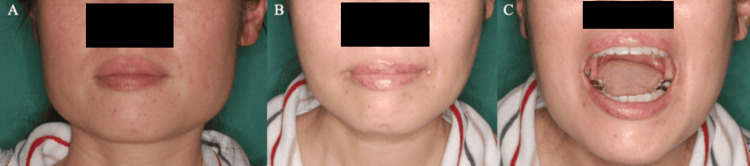
Photographs of faces (A) Preoperative. (B) Postoperative (two weeks). (C) Postoperative mouth opening range, showing an improvement from 25 mm to 53 mm.

Compared to the preoperative condition, the postoperative morphology of the mandibular angle is improved, and the mouth opening range is maintained following mandibular anglectomy (Figure 5). For mouth opening measurements, the forced maximum mouth opening distance was recorded at each surgical step, with the final measurement termed the “intraoperative mouth opening distance.” This distance was measured between the upper and lower incisors using a hexagonal mouth opening meter (Figure 3D). Postoperatively, patients were instructed to rest immediately after surgery and to begin mouth opening exercises approximately one week later. These exercises, performed three times daily with a wooden mouth opener, required patients to maintain the intraoperative mouth opening distance for 30 seconds. Follow-up assessments were conducted to measure the maximum unassisted mouth opening distance at one and six months post-surgery.

Statistical analysis

The Mann-Whitney U test was used for statistical analysis, with statistical significance set at p < 0.05.

## Results

The study included four males and 10 females, with an age range of 17-50 years (mean age: 33.9 years). The average preoperative mouth opening range under general anesthesia and muscle relaxation was 23.7 mm (range: 15-27 mm). Following surgery, the average intraoperative mouth opening range improved to 50 mm (range: 30-61 mm), and at one month postoperatively, it was 35.4 mm (range: 24-44 mm). The maximum intraoperative mouth opening range was 47.1 mm (range: 30-60 mm) in nine patients who underwent masseter muscle and temporalis tendonotomies, and 55.2 mm (range: 48-61 mm) in five patients who underwent mandibular anglectomy in addition to masseter and temporalis tendonotomies. The difference in opening range was significant (p = 0.0112).

At six months postoperatively, the mean mouth opening range in all patients was 40.1 mm (range: 20-53 mm). The maximum mouth opening range was 36.4 mm (range: 20-48 mm) in nine patients who underwent masseter and temporalis tendonotomies, and 46.8 mm (range: 41-53 mm) in five patients who underwent mandibular anglectomy, including masseter and temporalis tendonotomies. The difference in opening range at six months was significant (p = 0.0107).

**Figure 6:**
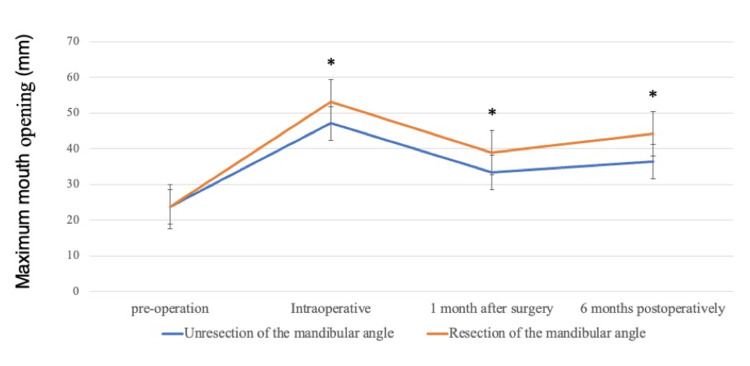
Maximum mouth opening This figure shows the maximum mouth opening range measured preoperatively, intraoperatively, one month postoperatively, and six months postoperatively (mm), along with the average value of the maximum mouth opening range (mm). The asterisk (*) indicates statistical significance (p ≤ 0.05).

In all cases, the mouth opening range was significantly greater than preoperatively. Mandibular anglectomy was effective in securing the mouth opening range. Patients who underwent mandibular anglectomy maintained their mouth opening range for six months postoperatively, whereas those who did not undergo mandibular anglectomy showed a decreasing trend in mouth opening range (Figure 6).

## Discussion

Regarding the choice of surgical approach, the authors prioritize bilateral masseter muscle tendonectomy and perform temporalis tendonotomy after achieving a certain degree of improvement in the mouth opening range. One reason for this approach is to assess whether the temporalis tendon or the muscular process is contributing to the opening disturbance. Additionally, performing temporalis tendonotomy is easier once the mouth opening range is increased. While MRI can offer some preoperative insights, we believe that intraoperative judgment remains crucial. If sufficient mouth opening can be achieved during surgery, mandibular anglectomy is unnecessary.

When mandibular anglectomy is combined with masseter tendonotomy and temporalis tendonotomy, the masseter muscle is detached from the mandibular branch, and the medial pterygoid muscle, attached to the inferior border of the mandible, is also separated intraoperatively. This reduces resistance to mouth opening, allowing for a greater range of motion. Additionally, when mandibular anglectomy is performed, the resistance from the masseter and medial pterygoid muscles attached to the inferior border of the mandible is further reduced. The mean mouth opening range at six months postoperatively was 36.4 mm in nine patients who underwent masseter tendonotomy and temporalis tendonotomy, and 46.8 mm in five patients who underwent mandibular anglectomy combined with these procedures. These results suggest that mandibular angle reconstruction plays a significant role in maintaining mouth opening. Based on our findings, we have established a treatment goal of achieving at least 45 mm of mouth opening.

Patients with markedly distended mandibular angles often report pain in and around the mandibular angle during postoperative mouth opening training, which can restrict their mouth opening. If the mandibular angle compresses surrounding tissues during intraoperative mouth opening, mandibular anglectomy is an effective way to improve the mouth opening range. Furthermore, masseter tendonectomy and temporalis tendonotomy alone may create significant resistance to mouth opening. In such cases, removing the temporalis tendon attached to the medial oblique line of the mandibular branch, the masseter muscle from the mandibular branch, and the medial pterygoid muscle attached to the inferior border of the mandible can significantly reduce resistance. These results suggest that the masseter and medial pterygoid muscles, along with the hyperplasia of their tendons and tendinous structures, are responsible for the decreased extensibility of these muscles.

Overall, surgical methods should be selected and combined according to the patient’s condition, including the masseter, temporalis, and medial pterygoid muscles, as well as the mandibular angle. Additionally, a minimally invasive surgical approach should be prioritized based on long-term postoperative outcomes.

Although some cases show improvement with mouth opening training alone, it is not a definitive treatment and may lead to regression or failure to achieve satisfactory mouth opening. Maintaining the mouth opening range postoperatively is critical, and the prognosis often depends on effective training. For this reason, we consider it essential to initiate mouth opening training before surgery. This helps patients understand the importance of postoperative mouth opening training and teaches them the techniques needed for self-training. Ultimately, surgical treatment is the standard therapy, and the long-term results are typically satisfactory. Conservative treatments alone, such as pharmacotherapy, occlusal splints, and physical therapy, are generally ineffective [10,14,15].

Postoperatively, in our hospital, we maintain closed-mouth rest with compression bandages immediately after surgery and begin mouth opening training within one week. Early initiation of postoperative mouth opening training is critical for maintaining the mouth opening range. In our experience, patients who did not engage in adequate postoperative training showed a decrease in mouth opening range. Although mouth opening training methods vary across institutions, most begin training between one and seven days after surgery, continuing daily for an extended period. The most important factor in postoperative therapy is the patient’s commitment to mouth opening training. The most effective methods include the use of hands and assistive devices, such as wooden mouth openers used in our hospital.

The results of this study provide insights into the surgical treatment methods for masticatory muscle tendon and tendon membrane hyperplasia at our hospital, confirming the effectiveness of mandibular anglectomy. However, the sample size was small, and this study was conducted at a single hospital. We anticipate that as the number of MMTAH cases increases, multi-institutional collaborative studies will provide further insights. Additionally, mandibular anglectomy was found to be particularly useful for maintaining mouth opening at six months postoperatively. Since the mouth opening range does not improve beyond the intraoperatively achieved range, mandibular anglectomy could offer a better prognosis if the intraoperative mouth opening range is small. We recommend that intensive training during the first month after surgery is vital and that emphasizing the importance of preoperative mouth opening training will help patients maintain their mouth opening range postoperatively. Given the limited number of reports on MMTAH in the literature, further research will be essential to better understand its pathophysiology and to develop improved treatment strategies for this condition.

## Conclusions

This study examined the current status and effectiveness of surgical treatment for MMTAH, with a particular focus on mandibular angle resection as an effective approach for maintaining the mouth opening range. Mandibular angle resection has proven to be a valuable surgical procedure for preserving postoperative quality of life, and we have found that it can be safely performed with the use of a cutting guide. However, given the limited number of cases in this study, conducted at a single institution, and the rarity of MMTAH, we advocate for further accumulation of cases and the initiation of randomized multicenter prospective studies with long-term follow-up to better evaluate the outcomes and refine treatment strategies.
